# Genetic mapping of anthocyanin accumulation-related genes in pepper fruits using a combination of SLAF-seq and BSA

**DOI:** 10.1371/journal.pone.0204690

**Published:** 2018-09-27

**Authors:** Guoyun Wang, Bin Chen, Heshan Du, Fenglan Zhang, Haiying Zhang, Yaqin Wang, Hongju He, Sansheng Geng, Xiaofen Zhang

**Affiliations:** Beijing Vegetable Research Center, Beijing Academy of Agriculture and Forestry Sciences, Key Laboratory of Biology and Genetic Improvement of Horticultural Crops (North China), Ministry of Agriculture, Beijing, P.R. China; NARO Institute of Fruit Tree Science, JAPAN

## Abstract

Anthocyanins have significant functions in stress tolerance in pepper (*Capsicum annuum* L.) and also benefit human health. Nevertheless, the key structural genes and regulatory genes involved in anthocyanin accumulation in pepper fruits are still not well understood and fine mapped. For the present study, 383 F_2_ plants from a cross between the green-fruited *C*. *annuum* line Z5 and the purple-fruited line Z6 was developed. Two separate bulked DNA pools were constructed with DNAs extracted from either 37 plants with high anthocyanin content or from 18 plants with no anthocyanin. A combination of specific-locus amplified fragment sequencing (SLAF-seq) and bulked segregant analysis (BSA) was used to identify candidates for regions associated with anthocyanin accumulation. We identified a total of 127,004 high-quality single nucleotide polymorphism (SNP) markers, and detected 1674 high-quality SNP markers associated with anthocyanin accumulation. Three candidate anthocyanin-associated regions including the intervals from 12.48 to 20.00 Mb, from 54.67 to 56.59 Mb, and from 192.17 to 196.82 Mb were identified within a 14.10-Mb interval on chromosome 10 containing 126 candidate genes. Based on their annotations, we identified 12 candidate genes that are predicted to be associated with anthocyanin expression. The present results provide an efficient strategy for genetic mapping of and valuable insights into the genetic mechanisms of anthocyanin accumulation in pepper fruit, and allow us to clone and functionally analyze the genes that influence anthocyanin accumulation in this species.

## Introduction

Anthocyanins are soluble flavonoid plant pigments that confer colors ranging from bright red-orange to blue-violet or black [1] to fruits, flowers, seeds, leaves, and stems. Anthocyanins are involved in pigmentation, attraction of seed distributors and pollinators [2, 3], and protection against photo-oxidative damage in plants [4], and might have health-promoting effects in humans [5, 6]. Anthocyanins are accumulated in the palisade and mesophyll cells of purple or black leaves and in the outer mesocarp of black and violet fruit [1]. The violet or black pigmentation of pepper is also due to high levels of anthocyanin [1, 7]. Therefore, new pepper cultivars with high anthocyanin content could both improve stress tolerance in pepper plants and enhance health benefits for humans.

Extensive studies of the biosynthetic pathway leading to anthocyanins have found 12 structural genes and three transcription factors (TFs) involved in the pathway [8–10]. Anthocyanin biosynthesis begins in the phenylpropanoid pathway followed by the flavonoid pathway (S1 Fig) [8]. Phenylpropanoid pathway enzymes phenylalanine ammonia-lyase (PAL), cinnamate 4-hydroxylase (C4H), and 4-coumarate CoA ligase (4CL) transform phenylpropanoid into 4-coumaroyl-CoA. In the flavonoid pathway, the product of condensation of one 4-coumaroyl-CoA with three malonyl-CoA is converted into dihydroflavonol by the enzymes chalcone synthase (CHS), chalcone isomerase (CHI), and flavanone 3-hydroxylase (F3H). Dihydroflavonol is then transformed into dihydromyricetin, a colorless molecule, by the enzyme flavonoid 3´,5´-hydroxylase (F3´5´H). This compound is subsequently reduced by dihydroflavonol 4-reductase (DFR) and ultimately converted into blue delphinidin by anthocyanidin synthase (ANS). All other anthocyanins are then derived from delphinidin. The predominant anthocyanin in pepper (*C*. *annuum* L.) is delphinidin-3-*trans*-coumaroylrutinoside-5-glucoside [10–12]. The delphinidins undergo further modification *via* glycosylation catalyzed by UDP glucose-flavonoid 3-O-glycosyl-transferase (UFGT/3GT), 5/7-O-glycosyl- transferases (5GT/7GT) [13, 14], rhamnosylation catalyzed by rhamnosyl transferases (RTs) [15], acylation catalyzed by acyl transferases (AT) [16], and methylation catalyzed by methyltransferases (MTs) [17]. The anthocyanins are subsequently transported into the vacuole, which might be mediated by glutathione S-transferase (GST) [18, 19], anthocyanic vacuolar inclusions (AVIs) in the cytoplasm [20], and anthocyanin permease (ANP) [8, 9]. A series of structural genes that encode these anthocyanin biosynthetic enzymes have already been cloned. Each of these genes is expressed in a specific way depending on the tissue or developmental stage. The sequences of these genes are also highly conserved across plant species [8, 10].

A regulatory complex comprised of the basic helix-loop-helix (bHLH) protein R2R3-MYB plus WD40 repeats (MYB-bHLH-WD40) interacts with structural gene promoters to modulate the expression of anthocyanin structural genes [7, 12, 21, 22]. Among these, MYB appears to be an important determinant in anthocyanin accumulation [10]. MYB and WD40 TFs control the expression of some late (*F3’5’H*, *DFR*, or *3GT*) and early anthocyanin biosynthesis pathway structural genes (*CHS*, *CHI*, or *F3H*) in pepper fruits [9]. However, this result differs from that of Borovsky et al. (2004), which found that only the expression of the late structural genes (*DFR*, *ANS*) is dependent on the incompletely dominant gene *anthocyanin* (*A*) [15]. The *A* locus, which encodes a MYB TF (CaMYB), controls the expression of anthocyanin in various tissues of pepper [15, 23–25]. Li et al. (2013) also found that *CaMYB1* and *CaMYB2* which were homologous to *CaMYB* might regulate the expression of anthocyanin biosynthesis in green fruits of hot pepper [26]. Ben-Chaim et al. (2003) mapped the pepper *A* locus, which is homologous to tomato *anthocyanin 1* (*ANT1*) and petunia *anthocyanin 2* (*AN2*) [15], onto pepper chromosome 10 [24]. In addition, the *A* locus is linked to a major-effect quantitative trait locus (QTL) (*fs10*.*1*) controlling pepper fruit shape [24, 27]. Borovsky and Paran (2011) mapped *fs10*.*1* to 0.3 cM from the its nearest molecular marker, *CT11*, and 6.3 Mb from *ANT1*, which is the ortholog of the pepper *A* gene in tomato, according to the tomato genome assembly release 2.30 (http://solgenomics.net/) [27]. The pepper genome sequence was published in 2014 [28], so the physical distance between *fs10*.*1* and *CT11* in pepper was not known at that time. Informative, saturated linkage maps are important for fine mapping quantitative traits. Due to the lack of saturated linkage maps in pepper, the anthocyanin accumulation trait had also not yet been finely mapped in this species.

The genome sequence of pepper [28] will enable fine mapping of anthocyanin accumulation and other traits in pepper. Efficient identification of genes or QTL linked to plant traits of interest will become possible by combining specific-locus amplified fragment sequencing (SLAF-seq) with bulked-segregant analysis (BSA) [29–34]. SLAF-seq is a type of next-generation, reduced-representation genome sequencing strategy for efficient identification of single-nucleotide polymorphisms (SNPs), while BSA allows subsequent rapid screening of bulked pools of DNA to identify molecular markers linked closely to the target allele of a gene or QTL controlling a trait of interest [35, 36].

Here, BSA combined with SLAF-seq was first time to be used to map the anthocyanin accumulation trait in pepper by pooling DNAs from F_2_ plants with distinct anthocyanin accumulation phenotypes from a cross between the inbred *C*. *annuum* lines Z5 and Z6 as parents. The purple pepper line Z6 has high anthocyanin content with purple stems and fruit, while the green pepper line Z5 has no anthocyanin with green stems and fruit. Although a lot of anthocyanin-related genes have been isolated so far, but only *A* locus in pepper has been mapped. Nevertheless, the anthocyanin accumulation-related genes including *A* locus have not been fine mapped due to the lack of saturated linkage maps in pepper and candidate genes for bHLH, WD40 and variation of anthocyanin have not been identified. Thus, the main objective of this study was to identify genome regions and candidate genes related to anthocyanin accumulation-related genes in pepper, and to develop SNP markers to provide diagnostic SNP markers for molecular marker-assisted selection of anthocyanin accumulation genes and lay a foundation for cloning.

## Materials and methods

### Plant materials

We used the green-fruited *C*. *annuum* line Z5 and purple-fruited *C*. *annuum* line Z*6* as parental lines in the present study. Z6 is an anthocyanin-pigmented variety (0.96 mg anthocyanin/100 g fresh weight) that has purple stems and fruit, while Z5 contains no anthocyanins and has green stems and fruit (S1 Table). We obtained an F_1_ hybrid from a Z5 × Z6 cross, and developed the F_2_ mapping population of 383 plants by self-pollinating the F_1_ hybrid. The parental plants, and F_1_ and F_2_ populations were grown in a greenhouse at Beijing Vegetable Research Center, Beijing Academy of Agriculture and Forestry Sciences, Beijing, China.

### Extraction and measurement of anthocyanin content

Pepper fruits were harvested at 20 d post-anthesis following the method of Aza-González et al. (2013) [8]. The pepper fruit pericarps were dissected and immediately immersed into liquid nitrogen, and the frozen pericarps were stored at -80 °C. Extraction and measurement techniques described in Lee et al. (2005) [37] were used to isolate and quantify anthocyanins in pericarp tissue. In brief, three aliquots of 20.00 g of frozen pulverized pericarp tissues were extracted in 100 mL of 0.1% (v/v) hydrochloric acid in methanol for 2 h at RT with lights on, and then were filtered. The filtered extract was then transferred to a 250-mL volumetric flask and the residue was re-extracted with hydrochloric acid in methanol at least three times until a spectrophotometrically colorless extract was obtained. Two aliquots of 50 mL of combined extracts were condensed to dryness in a rotary evaporator (Buchi, Flawil, Switzerland), and then were diluted with either 0.025 M potassium chloride (pH 1.0) or 0.4 M sodium acetate (pH 4.5) in separate 25-mL volumetric flasks. A test aliquot was then diluted to determine the dilution factor with pH 1.0 buffer until reaching an absorbance of 0.2 to 0.8 at 520 nm (A520). The absorbances at both 520 and 700 nm of test aliquots diluted with pH 1.0 or pH 4.5 buffer were then measured. The anthocyanin concentration as cyanidin-3-glucoside equivalents was calculated as: Anthocyanin (cyanidin-3-glucoside equivalents, mg/L) = $\frac{A\times \mathrm{MW}\times \mathrm{DF}\times 10^{3}}{\varepsilon \times 1}$, where A = (A520nm–A700nm)_pH 1.0_ –(A520nm–A700nm)_pH 4.5_; MW (the molecular weight of cyanidin-3-glucoside) = 449.2 g/mol; DF = the dilution factor determined above; l = cm path length; ε = molar extinction coefficient of cyanidin-3-glucoside; and 10^3^ convert from g to mg.

### Extraction and pooling of DNA

Young leaves were harvested from both parents and F_2_ plants and genomic DNA was isolated using the N-cetyl N,N,N-trimethylammonium bromide (CTAB) method [38]. The two DNA pools for bulked segregant analysis (BSA) were constructed by combining equal amounts of DNA from high- or low-anthocyanin F_2_ plants chosen based on measured anthocyanin concentrations (S1 Table). The high-anthocyanin pool (H-pool) was comprised of DNA from 37 plants with high anthocyanin content that ranged from 0.90 to 7.31 mg per 100 g fresh weight, and the low-anthocyanin pool (N-pool) was comprised of DNA from 18 plants with no measurable anthocyanin content. DNA isolated from parental lines Z5 and Z6 and the two pools of F_2_ DNA were used for construction and sequencing of SLAF libraries.

### Construction and high-throughput sequencing of SLAF libraries

An initial restriction enzyme digestion experiment was performed in the pepper line CM334 to select the appropriate restriction enzyme for SLAF library construction as per Xu et al. (2015) [39]. *Rsa*I (New England Biolabs, Ipswich, MA, USA) was chosen for digesting the parental and bulked genomic DNAs because it resulted in even distribution of SLAFs on each pepper CM334 chromosome (S2 Fig). Single-nucleotide A overhangs were added to the resulting DNA fragments using Klenow fragment (USB, Cleveland, OH, USA), and then fragments were ligated with dual-index sequencing adaptors [40] and amplified by PCR (polymerase chain reaction). Amplified fragments were then purified, pooled, and screened for the optimal fragment size range for construction of SLAF libraries, as described by Sun et al. (2013) [36] with minor modifications. DNA fragments of 364 to 414 bp were identified and subjected to paired-end sequencing to identify SLAFs using the Illumina HiSeq 2500 platform (Illumina, Inc., San Diego, CA, USA) at Beijing Biomarker Technologies Corporation (http://www.biomarker.com.cn).

### Evaluation for the SLAF libraries

To validate sequencing procedures and accuracy, we used rice (*Oryza sativa* L. *japonica*) as a control with the version 7.0 rice reference genome (http://rice.plantbiology.msu.edu/) with the same library construction and sequencing methods as used for the Z5 × Z6 mapping population to evaluate the SLAF libraries constructed for pepper. First, BWA software was used to compare the rice control sequencing reads with those of the pepper reference genome [41]. As Table 1 shows, we achieved a typical 84.94% efficiency of mapping paired-end reads in the rice control. Because complete digestion by a restriction enzyme improves SLAF experiments, enzyme digestion efficiency was evaluated as an index of optimal SLAF results, which can be affected by genome complexity, DNA purity, and restriction enzyme digestion completeness. Second, the efficiency of digestion of rice genomic DNA in the present study was 93.81% (Table 1), which was adequate for construction of these SLAF libraries. Finally, the read lengths of the paired-end reads of the rice control that mapped to the rice reference genome ranged from 364 to 414 bp (S3 Fig). These results indicated the construction of SLAF libraries were accurate.

**Table 1:** Efficiency of mapping paired-end reads and enzyme digestion in the rice control.

Mapped reads	Efficiency (%)	Enzyme digestion	Efficiency (%)
Mapped paired-end reads	84.94	Complete digestion	93.81
Mapped single-end reads	4.58	Partial digestion	6.19
Unmapped reads	10.48	—	—

### Analysis of SLAF-seq data

Raw sequence reads from each sample generated on the Illumina HiSeq 2500 platform were subjected to a series of quality control procedures including assessment of sequence quality scores and guanine-cytosine (GC) content to ensure reliable and unbiased reads [36]. More than 80% of sequences in the four libraries had quality scores greater than or equal to Q30 (where a quality score of Q30 indicates 0.1% error probability, or a 99.9% probability of sequence accuracy). We used BWA software to align clean sequence reads against the *C*. *annuum* cv. Criollo de Morelos 334 reference genome CM334 (http://peppergenome.snu.ac.kr/download.php, version 1.55) [28, 41]. BLAT [42] was then used to cluster all paired-end reads that had distinct index data by sequence similarities among the two parents and the pooled libraries. Sequences that were over 90% identical were then considered as a single SLAF locus or tag.

### Detecting high-quality SNPs

We used GATK [43] and SAMtools [44] software to detect single-nucleotide polymorphisms (SNPs). We designated the intersection of SNPs detected by both GATK and SAMtools as the ultimate set of SNPs to subject to further analysis. SnpEff software [45] was then used to annotate whether SNPs were located upstream or downstream from a nearby gene, or in an intergenic region, and whether they were synonymous or non-synonymous mutations with reference to annotated gene models from the pepper reference genome. SNPs were filtered prior to association analysis according to the following criteria: SNPs with multiple alleles were excluded; SNPs with sequencing depth of less than 4-fold in each F_2_ pool or parental DNA were omitted; SNPs with identical genotypes between pools were eliminated; and SNPs exhibiting recessive alleles that were not inherited from the recessive parent were excluded. Ultimately, the resulting high-quality SNPs were then used in association analysis.

### Association analysis

Both Euclidean distance (ED) [43] and a SNP-index algorithm [46, 47] were used to conduct the association analysis. The ED of the allele frequencies of each SNP was calculated between the H-pool and N-pool, as described by Hill et al. (2013) [43]: $ED=\sqrt{{\left(A_{N-pool}-A_{H-pool}\right)}^{2}+{\left(C_{N-pool}-C_{H-pool}\right)}^{2}+{\left(G_{N-pool}-G_{H-pool}\right)}^{2}+{\left(T_{N-pool}-T_{H-pool}\right)}^{2}}$, where *A*, *C*, *G*, or *T* refers to the frequency of each nucleotide. Euclidean distances were squared (ED^2^) to reduce noise and increase the effect of large ED values. The ED values from the H-pool and N-pool were fitted using a Loess regression analysis [43], and the significance threshold for marker-trait associations was set at three standard deviations above the median of the Loess-fitted values. Regions with Loess-fitted values exceeding this threshold were considered as the candidate genomic regions associated with the anthocyanin content of pepper in this cross.

The SNP-index algorithm is a useful tool for identifying significant between-pool differences in genotype frequencies. We used SLAF depth to represent genotype frequency in our SNP-index algorithm [48]. We calculated the ΔSNP-index as follows: ΔSNP-index = SNP-index (H-pool)–SNP-index (N-pool), where SNP-index (N-pool) = N_Np_/(N_Np_ + H_Np_) and SNP-index (H-pool) = H_Hp_/(H_Hp_ + N_Hp_). N_Np_ and H_Np_ indicate the depth of the N-pool derived from Z5 and Z6, respectively; and N_Hp_ and H_Hp_ represent the depth of the H-pool derived from Z5 and Z6, respectively. Thus, the ΔSNP-index equals zero if the SNP-indices of the N-pool and the H-pool are equal. A ΔSNP-index closer to 1 suggests that high anthocyanin content is almost completely associated with one genotype, and that the associated SNPs are closely linked to genomic regions conferring high anthocyanin content. In contrast, a ΔSNP-index closer to –1 indicates that the associated SNPs are linked to genomic regions conferring low anthocyanin content. We calculated the confidence coefficient for each ΔSNP-index then fitted ΔSNP-index values using the SNPNUM method [44]. The genomic regions associated with a trait are identified when the fitted marker values are greater than the 99% confidence coefficient threshold.

Ultimately, the intersections of candidate regions identified by both the ED and SNP-index approaches were designated as the final candidate anthocyanin-related regions. We then plotted ED, SNP-index, and ΔSNP-index data individually, and used CIRCOS 0.66 (http://circos.ca/) to plot a circular graph of the distributions of chromosomes, genes, SNPs, ED values, and ΔSNP-indices.

### Candidate gene annotation and screening

The functional annotations of candidate genes were explored using blastx alignment with default parameters to sequences at the Cluster of Orthologous Groups of proteins database (COG, http://www.ncbi.nlm.nih.gov/COG/), Gene Ontology (GO, http://www.geneontology.org/), the Kyoto Encyclopedia of Genes and Genomes (KEGG, http://www.genome.jp/kegg/), the NCBI non-redundant protein database (NR, ftp://ftp.ncbi.nih.gov/blast/db/), and Swiss-Prot (http://www.uniprot.org/). All genes that could be related to the synthesis, storage, or regulation of anthocyanin accumulation, as well as any genes containing SNPs resulting in non-synonymous mutations were chosen for further analysis as candidate genes.

## Results

### Analysis of sequence data and identification of SNPs

**Fig 1:**
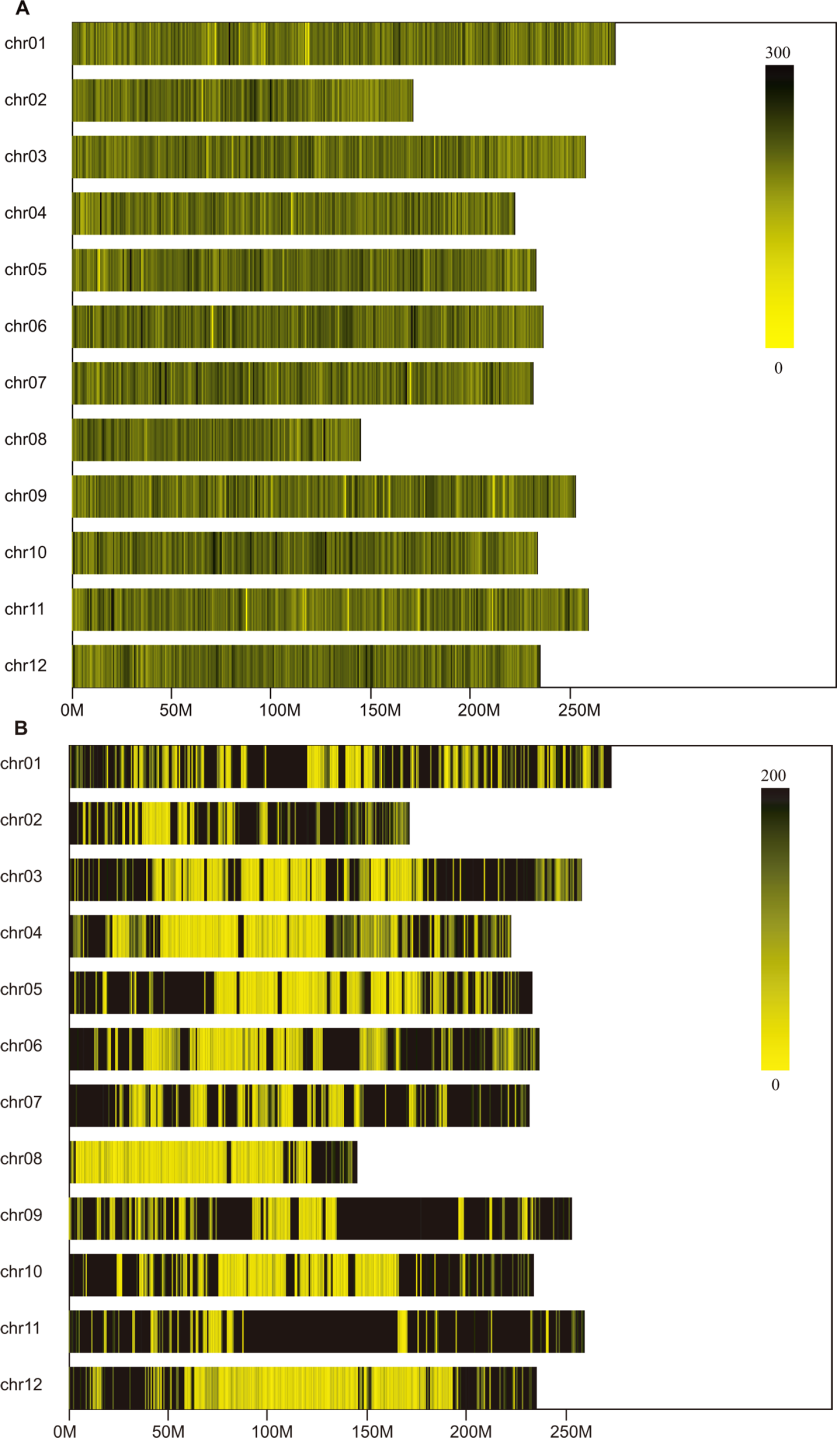
Distribution of SLAFs and SNPs on each chromosome of pepper. The *x*-axis and *y*-axis represent chromosome length and chromosome number, respectively. The distance between two adjacent yellow bars indicates 1 Mb on the chromosome, and black lines indicate SLAFs or SNPs.

A total of 52.76 Mb and 27.65 Mb of clean sequence reads with an average read length of 50 bp were obtained in the SLAF libraries for the two pools and parents, respectively (Table 2). The average percentage of Q30 bases was 94.50% and the GC content was 37.03%, indicating a majority of high-quality bases. Using BWA, we could map over 90.81% of the clean sequence reads to the CM334 pepper reference genome, indicating the level of sequencing accuracy. After clustering, 771,025 SLAFs were evenly distributed on the CM334 pepper reference chromosomes (Fig 1A; S2 Table). The numbers of SLAFs ranged from 41,197 on chromosome 08 to 73,838 on chromosome 01, 603,960 from the N-pool, 556,567 from the H-pool, 491,731 from Z6, and 496,868 from Z5. The average SLAF sequencing depths were 23.45-, 25.77-, 33.83-, and 45.74-fold in Z6, Z5, the H-pool, and the N-pool, respectively.

**Table 2:** Sequence data summary for parental lines Z5 and Z6 and bulked H-pool and N-pool.

Sample	Clean_Reads	Q30 (%)	GC (%)	Mapped reads rate (%)	Number of SLAFs	Total depth	Average depth
N-pool	31,297,954	94.76	36.70	90.85	603,960	27,624,290	45.74
H-pool	21,460,775	94.35	37.11	90.50	556,567	18,829,026	33.83
Z6	13,090,095	94.37	37.20	91.11	491,731	11,529,003	23.45
Z5	14,556,569	94.50	37.12	90.77	496,868	12,806,096	25.77

N-pool, the library created from pooled DNA from plants with no anthocyanin content; H-pool, and the library created from pooled DNA from plants with high anthocyanin content; Mapped reads rate (%), the proportion of the clean sequence reads mapped to the CM334 reference genome relative to the total clean reads; Q30, a quality score of 30 indicates 0.1% error probability or 99.9% sequence accuracy; GC, guanine-cytosine content; SLAF, specific-locus amplified fragment.

GATK [43] and SAMtools [44] software were used to identify a total of 836,852 SNPs, whose properties are shown in S3 Table. The most SNPs, 127,688, were detected on chromosome 12, while the fewest, 17,073, were detected on chromosome 08 (S2 Table). These SNPs were mapped to each chromosome on the CM334 reference genome as shown in Fig 1B. A total of 127,004 high-quality SNPs remained after filtering for use in the association analysis to find candidate anthocyanin-associated regions in pepper.

### Association analysis of anthocyanin content

**Fig 2:**
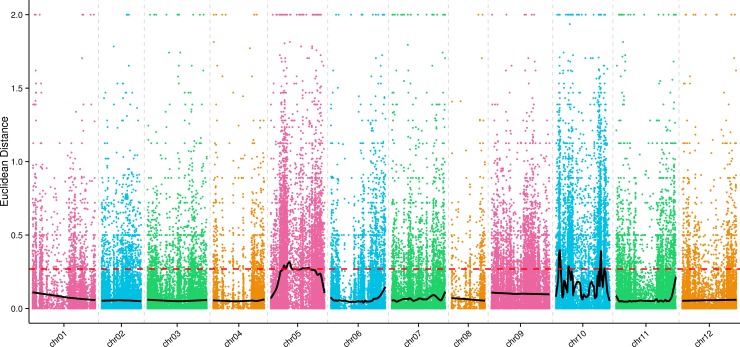
Association values for anthocyanin accumulation on each pepper chromosome from Euclidean distance-based association analysis. The 12 pepper chromosomes are represented along the *x*-axis, and the association values based on Euclidean distance (ED) are indicated along the *y*-axis. ED-based association values at each SNP location are represented by colored dots. The red dashed line represents the association threshold and black line indicates Loess-fitted values. When ED-based association values are higher, stronger association of a SNP with anthocyanin accumulation is indicated.

The ED value at each SNP locus was calculated for the 127,004 high-quality SNP markers. We squared ED values to reduce noise, and then fitted the association values by Loess regression analysis [43]. The threshold for association between markers and the anthocyanin trait was set to 0.27, or three standard deviations above the median of all the Loess-fitted values. When the Loess-fitted value was greater than 0.27, we detected three candidate regions on chromosome 05 for anthocyanin accumulation, and four candidate regions on chromosome 10 for this trait (Fig 2). As shown in Table 3, we identified 270 genes in a total interval of 80.12 Mb on chromosome 05. On chromosome 10, we identified 64 genes in the interval from 11,722,320 to 20,005,690 bp, 20 genes in the interval from 53,805,574 to 57,945,748 bp, 68 genes in the interval from 191,899,653 to 196,817,467 bp, and 15 genes in the interval from 210,028,131 to 211,321,646 bp. A total of 437 genes and 6268 high-quality SNP markers were found in these candidate regions, among which we identified two genes containing SNPs resulting in non-synonymous mutations. As shown in S4 Table, a total of 7537 and 2781 SNP markers were detected in parents and bulked pools, respectively, and more than 97.00% of these SNPs were annotated as located in the intergenic regions.

**Table 3:** Summary information for SNP-index and Euclidean distance-based association analyses.

ChrID	Start (bp)	End (bp)	Interval size (Mb)	Number of genes	Number of SNPs
**Euclidean distance**					
Chr05	53,667,767	93,840,115	40.17	124	3,238
Chr05	100,693,328	103,396,658	2.70	8	37
Chr05	132,573,706	169,821,807	37.25	138	891
Chr10	11,722,320	20,005,690	8.28	64	961
Chr10	53,805,574	57,945,748	4.14	20	341
Chr10	191,899,653	196,817,467	4.92	68	601
Chr10	210,028,131	211,321,646	1.29	15	199
**SNP-index**					
Chr10	12,479,910	20,005,690	7.53	52	912
Chr10	54,672,471	56,593,567	1.92	6	226
Chr10	192,166,533	196,817,467	4.65	68	536

Chr, chromosome; SNP, single-nucleotide polymorphism.

**Fig 3:**
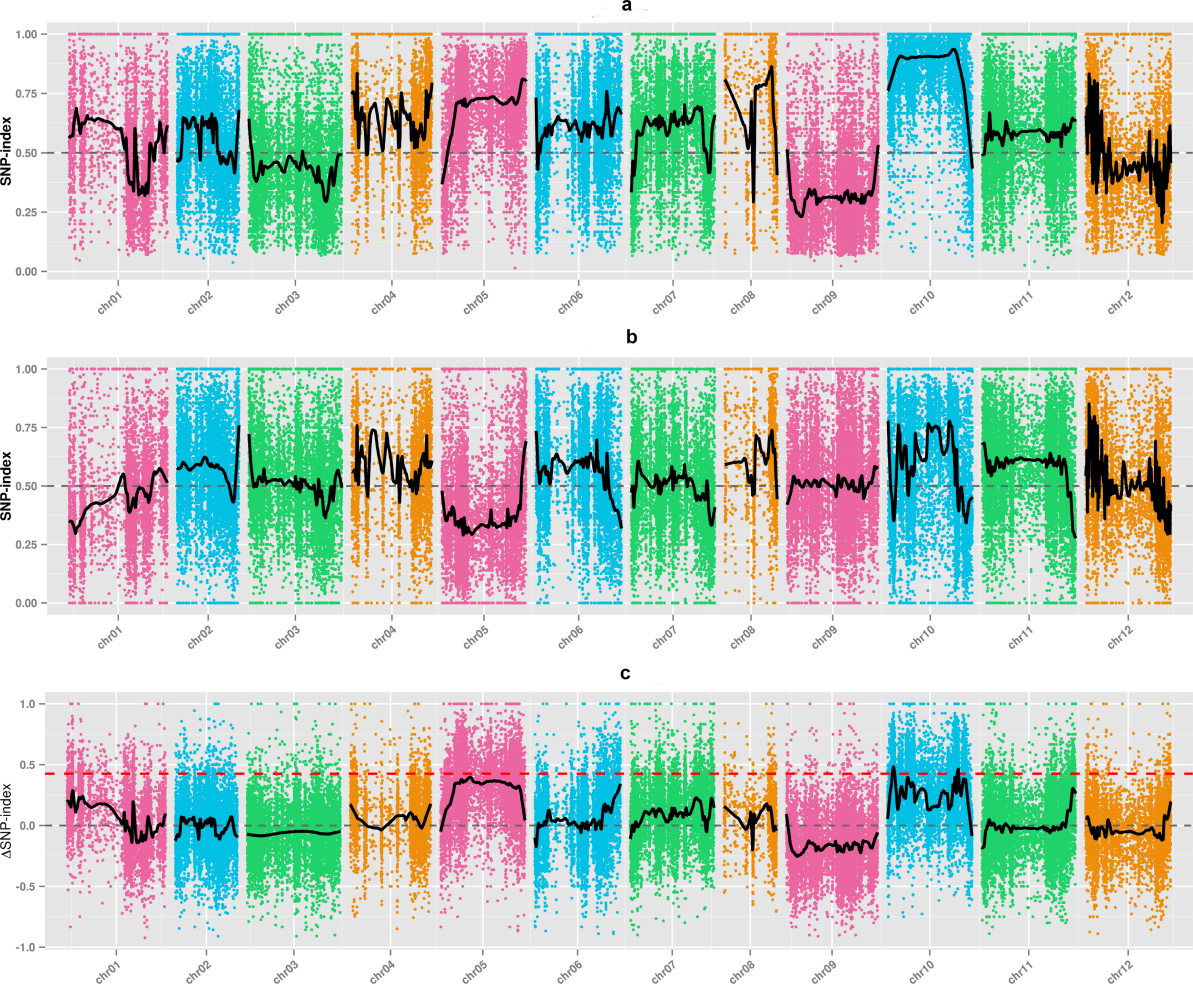
**Graphs of H-pool (a) and N-pool (b) indices and ΔSNP-index (c) for SNP-index-based association analysis**. The 12 chromosomes are represented along the *x*-axis, and the SNP index or ΔSNP-index values are shown along the *y*-axis. The black line indicates fitted SNP-index or ΔSNP-index values. The red, blue, and green lines indicate the thresholds at the 99%, 95%, and 90% confidence coefficients, respectively.

ΔSNP-index values were determined by subtracting the H-pool SNP-index from the N-pool SNP-index. Average SNP-indices for the H-pool and N-pool were plotted in 400-SNPsliding windows in 1-SNP steps in the CM334 genome assembly. The SNP-indices of the H- and N-pools and the ΔSNP-index are shown plotted in Fig 3A, Fig 3B, and Fig 3C, respectively. When the fitted SNP marker values exceeded the threshold at a 99% confidence coefficient, three candidate regions for anthocyanin accumulation were detected on chromosome 10 by examining ΔSNP-index values (Fig 3C; Table 3). There were 52 genes in the intervals from 12,479,910 to 20,005,690 bp, six genes in the interval from 54,672,471 to 56,593,567 bp, and 68 genes in interval from 192,166,533 to 196,817,467 bp, or a total of 126 genes in a 14.10 Mb candidate region interval. One gene containing a SNP resulting in a non-synonymous mutation was found in the interval from 194,974,062 to 194,974,736 bp. In addition, 1674 high-quality SNP markers were identified and mapped in the candidate regions (Table 3), and 2069 and 926 SNP markers we identified between the parents and bulked H-pool and N-pool, respectively (S4 Table). A total of 97.20% of the SNP markers in parents and 96.54% of the SNP markers in bulked pools were annotated as located in intergenic regions.

Final candidate regions were determined by overlapping the ED and SNP-index association analysis results to identify regions tightly associated with anthocyanin content. The final candidate regions were the same as those identified by SNP-index association analysisin the intervals from 12.48 to 20.00 Mb, from 54.67 to 56.59 Mb, and from 192.17 to 196.82 Mb on chromosome 10.

### Visualization of results of genomic analysis of anthocyanin accumulation with the combined SLAF-seq/BSA strategy

To more clearly visualize all of the results from the present study, we plotted a circular graph (S4 Fig) representing, in order from the first circle to the fifth circle, the 12 chromosomes of pepper, the distributions of genes, SNP densities, ED values, and ΔSNP-index values.

### Annotation of SNPs and genes in the candidate anthocyanin- associated region

We detected a total of 2069 and 926 SNP markers in the candidate anthocyanin-associated genomic regions between the parents and between the bulked pools, respectively (S4 Table). Analysis using SnpEff software [45] indicated the 20 SNPs in the upstream regions of genes, 2011 SNPs in intergenic regions, and 26 SNPs in the downstream regions of genes between the parental lines. This analysis also revealed 10 SNPs in the upstream regions of genes, 894 SNPs in intergenic regions, and 13 SNPs in the downstream regions of genes between the bulked pools. Additionally, there were only two SNPs resulting in synonymous mutations and one SNP resulting in a non-synonymous in coding regions, indicating that most SNP markers were located in the intergenic regions (S4 Table).

**Fig 4:**
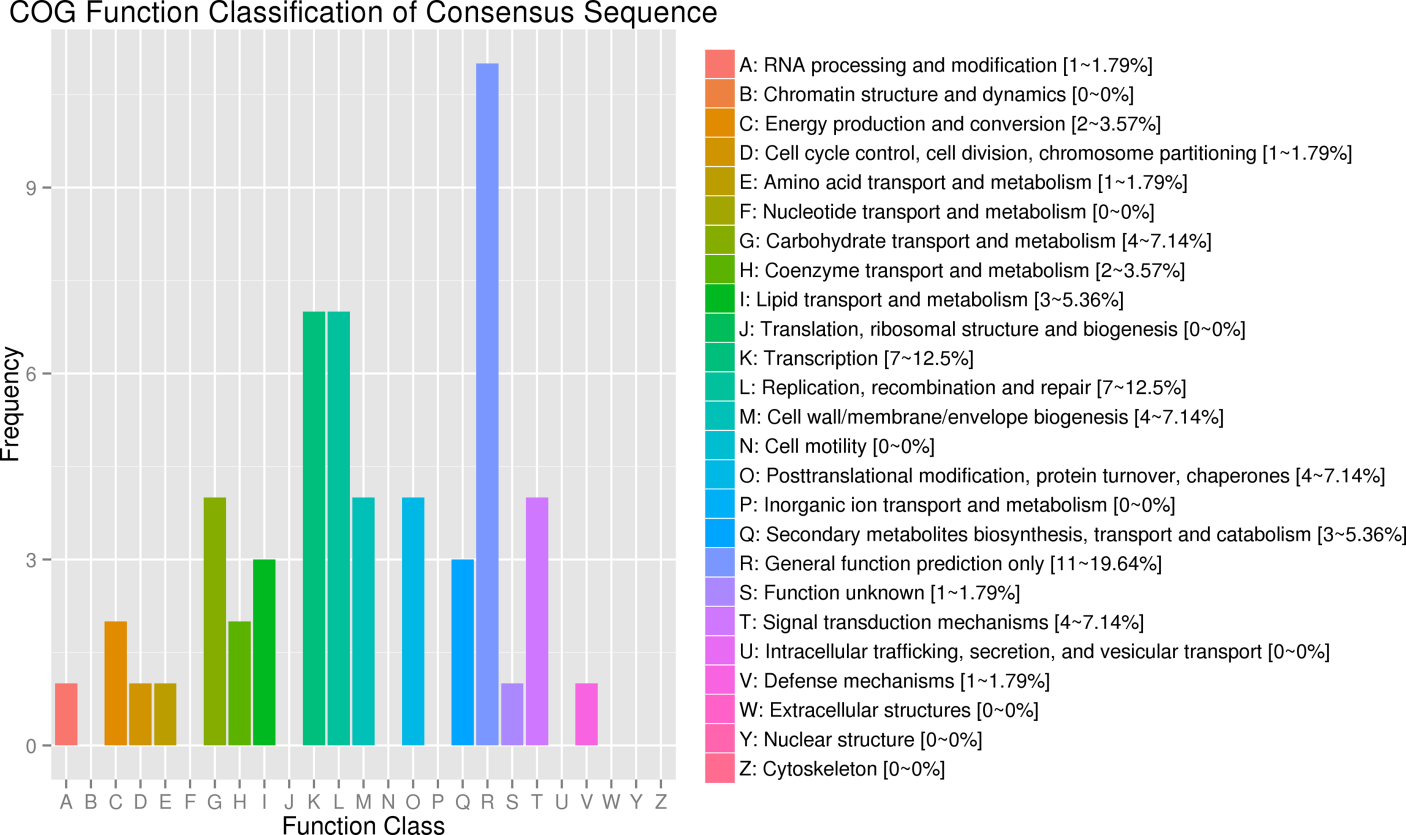
Functional annotations of candidate anthocyanin-associated genes according to the Cluster of Orthologous Groups of proteins database (COG). The x-axis indicates the classification annotation in COG and the y-axis represents the number of annotated candidate genes.

A total of 126 candidate genes related to anthocyanin content (S5 Table) were identified within the three candidate regions on chromosome 10 according to the current CM334 pepper reference genome annotation. Excluding 11 genes in the candidate regions that have not yet been annotated in public databases, we found annotations for 38, 101, 18, 100, and 126 genes within the candidate anthocyanin-associated regions in the COG, GO, KEGG, NCBI NR, and Swiss-Protdatabases, respectively (Table 4). The 38 genes predicted by COG analysis were classified by their putative functions (Fig 4); 11 genes were classified as ‘general function prediction only’ and seven genes were associated with ‘transcription’. The putative functions of seven other genes were classified as ‘replication, recombination and repair’. In addition, four genes were associated with ‘carbohydrate transport and metabolism’, ‘cell wall/membrane/envelope biogenesis’, ‘posttranslational modification, protein turnover, chaperones’, or ‘signal transduction mechanisms’, which accounted for 10.53% of all genes annotated in the COG analysis (S5 Table; Fig 4).

**Table 4:** Annotation statistics for candidate anthocyanin-associated pepper genes for anthocyanin accumulation.

Database	NR	GO	SwissProt	COG	KEGG	Total
Number of annotated	126	101	100	38	18	126
Number of CDS containing non-synonymous subsitutions	1	1	1	1	0	1

GO term enrichment analysis identified genes associated with enriched GO terms from the molecular function, biological process, and cellular component domains (S5 Fig). Because some genes had more than one annotation from different GO domains, genes could sometimes fall into more than one functional category (S5 Fig). The major enriched functional categories in our data included those associated with cellular components such as cell (GO:0005623), cell part (GO:0044464), or organelle (GO:0043226); molecular function such as catalytic activity (GO:0003824) or binding (GO:0005488); and biological process, specifically biological regulation (GO:0065007), cellular process (GO:0009987), metabolic process (GO:0008152), response to stimulus (GO:0050896), or single-organism process (GO:0044699). These results suggested that major metabolic changes in the biological process domain and posttranslational modifications in the molecular function domain might be involved in the regulation of anthocyanin content of pepper. At the same time, the regulation of anthocyanin content could also be associated with cell, organelle and cell part functions in the cellular component domain. Additionally, directed acyclic graphs (DAG) for GO terms were plotted using the Bioconductor package TopGO [49] to show the hierarchical parent-child relationships among GO terms (S6–S8 Figs). This analysis showed that the most-enriched term was chloroplast thylakoid membrane (GO:0009535) in the cellular component domain, DNA-directed RNA polymerase activity (GO:0003899) in the molecular function domain, and negative regulation of abscisic acid-activated signaling pathway (GO:0009788) in the biological process domain, which might be involved in the regulation of pepper anthocyanin content.

**Fig 5:**
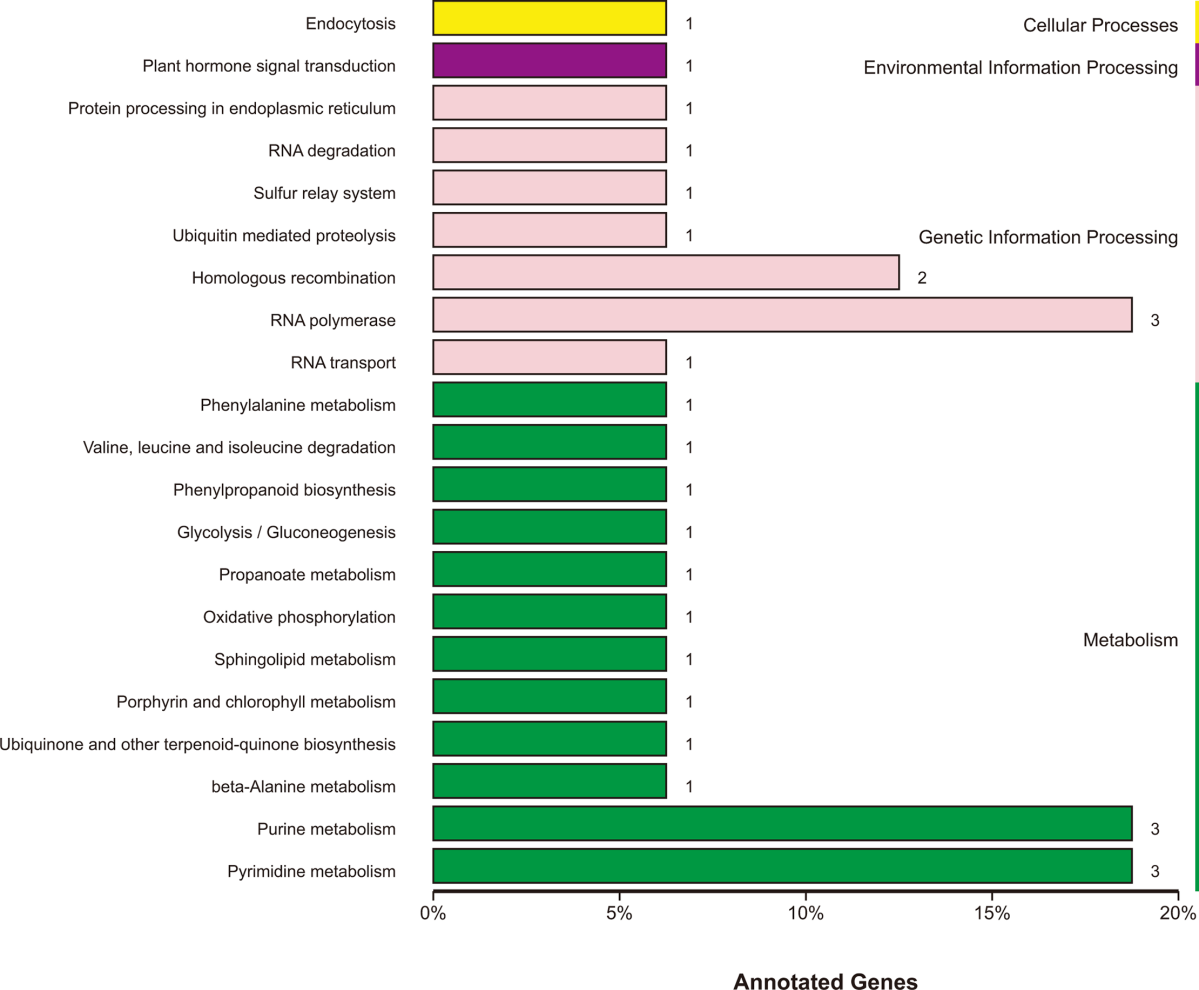
Pathways identified as enriched in the candidate regions via KEGG analysis. The *x*-axis represents the number and percentage of annotated candidate genes and the *y*-axis represents name of pathway in KEGG.

To better understanding of the functions of genes potentially involved in anthocyanin accumulation, we performed KEGG analysis and identified 18 genes in 22 pathways using KEGG analysis (Fig 5). One gene, *CA10g12720*, was involved in the ‘endocytosis’ pathway in cellular processes, and *CA10g12690* was involved in the ‘plant hormone signal transduction’ pathway that was associated with environmental information processing. In addition, 10 genes were identified in seven pathways in genetic information processing and 16 genes were identified in 12 pathways related to metabolism. Four pathways were identified as enriched (*P* <0.05) by KEGG enrichment analysis including more than two genes with predicted functions in the ‘RNA polymerase’ (ko03020), ‘homologous recombination’ (ko03440), ‘pyrimidine metabolism’ (ko00240), or ‘purine metabolism’ (ko00230) pathways (S6 Table) that are associated with the regulation of gene expression.

### Candidate genes correlated with anthocyanin accumulation

Annotations suggested that 12 candidate genes could have functions associated with anthocyanin accumulation in pepper (Table 5). Seven genes of these genes have annotations clearly associated with anthocyanin biosynthesis or metabolism. For example, *CA10g04060* is annotated as *4CL2* in the *4CL* gene family [50] and encodes a predicted 4-coumarate-CoA ligase 2 in the ‘ubiquinone and other terpenoid-quinone biosynthesis’ pathway (ko00130), or 4-coumarate-CoA ligase (GO:0016207) in the phenylpropanoid pathway. *CA10g03640* and *CA10g03760*, which encode a predicted anthocyanin 5-aromatic acyltransferase (*5AT*) and a predicted anthocyanidin 3-O-glucoside 6'-O-acyltransferase (*3AT*), respectively, were predicted to have anthocyanin 5-O-glucoside 6'-O-malonyltransferase activity (GO:0033810). *CA10g03880*, *CA10g12640*, and *CA10g12650* were all annotated as glucosyltransferases. *CA10g03880* was homologous to *3GGT* from *Ipomoea nil* in anthocyanin biosynthesis pathway [51]. *CA10g12640* and *CA10g12650* were predicted to encode proteins with flavonol 3-O-glucosyltransferase activity (GO:0047893). Additionally, *CA10g12890* (homologous to *Solanum melongena* cytochrome P450 76A2, *CYP76A2*) might encode a protein with *F3´5´H* activity (GO:0033772). Three out of 12 candidate genes were homologs of regulatory genes that regulate structural anthocyanin biosynthesis genes. *CA10g03650* was homologous to *MYB39* from *Arabidopsis thaliana*, which encodes a MYB39 TF involved in regulation of phenylpropanoid metabolism (GO:2000762). Meanwhile, *CA10g12810*, which is homologous to *Arabidopsis thaliana APL*, also encodes a MYB-family TF APL predicted to participate in the regulation of anthocyanin metabolism (GO:0031537). Further, Zhou et al. (2016) reported that *APL* was also involved in phenylpropanoid and flavonoid biosynthesis pathways [52]. *CA10g12710*, a homolog of *nol10* in *Danio rerio* was predicted to be a WD40 repeat protein based on its COG annotation. In addition, *CA10g03810* was homologous to *Arabidopsis thaliana* GDSL esterase/lipase At5g45960, which was associated with accumulation of anthocyanin in ultraviolet light-exposed plants (GO:0043481). Finally, *CA10g12840* was the only candidate gene containing a SNP resulting in a non-synonymous mutation. *CA10g12840* encodes a predicted subtilisin-like protease, which can bind to maltose binding protein [53] and, based on its COG annotation, is associated with ‘Posttranslational modification, protein turnover, chaperones’. The function of *CA10g12840* in anthocyanin accumulation needs to be further studied.

**Table 5:** Candidate genes related to anthocyanin accumulation in pepper.

Classification	Gene name	Gene_ID	Start (bp)	End (bp)	Description
Structural genes	*4CL2*	*CA10g04060*	18,570,738	18,572,196	4-coumarate—CoA ligase 2 *Nicotiana tabacum* [50]
	*5AT*	*CA10g03640*	12,745,342	12,746,112	Anthocyanin 5-aromatic acyltransferase *Gentiana triflora*
	*3AT*	*CA10g03760*	14,981,576	14,981,851	Anthocyanidin 3-O-glucoside 6”-O-acyltransferase *Perilla frutescens*
	*3GGT*	*CA10g03880*	16,367,650	16,369,032	Anthocyanidin 3-O-glucoside 2”-O-glucosyltransferase *Ipomoea nil* [51]
	*UGT73C3*	*CA10g12640*	194,040,338	194,041,813	UDP-glycosyltransferase 73C3 *Arabidopsis thaliana*
	*UGT73C5*	*CA10g12650*	194,053,311	194,053,472	UDP-glycosyltransferase 73C5 *Arabidopsis thaliana*
	*CYP76A2*	*CA10g12890*	195,250,338	195,253,243	Cytochrome P450 76A2 *Solanum melongena*
Transcription factors	*MYB39*	*CA10g03650*	13,533,765	13,535,787	Transcription factor MYB39 *Arabidopsis thaliana*
	*nol10*	*CA10g12710*	194,493,711	194,494,820	Nucleolar protein 10 *Danio rerio*
	*APL*	*CA10g12810*	194,789,125	194,791,433	MYB family transcription factor APL *Arabidopsis thaliana* [52]
Anthocyanin accumulation in response to stress	*At5g45960*	*CA10g03810*	15,633,033	15,636,114	GDSL esterase/lipase At5g45960 *Arabidopsis thaliana*
Genes with non-synonymous SNPs	*ARA12*	*CA10g12840*	194974062	194974736	Subtilisin-like protease *Arabidopsis thaliana* [53]

## Discussion

The pepper genome is large, with an estimated size of 3.48 Gb [28], and such large genomes can be relatively costly to analyze by low-coverage sequencing or whole genome deep resequencing. SLAF-seq is a relatively new strategy for genome-wide discovery of SNPs and genotyping on a large scale that can improve the throughput and accuracy of high-coverage sequencing, and also make it more efficient and cost effective [36]. A new strategy that combines SLAF-seq with BSA and takes advantage of both methods was used to analyze the genetic control of anthocyanin accumulation in the present study. This strategy also has been used for QTL analysis and linkage mapping in various species such as rice [29], cotton [30], and melon [32], as well as pepper [33, 34, 54]. For example, we previous had successfully mapped the pepper first flower node trait using the strategy, and the strategy had proven to be an effective method to identify candidate region and genes linked to a specific trait [54]. Anthocyanins are one of the determinants of pepper color, and also happen to increase abiotic stress tolerance in plants and have benefits for human health [4–6]. In pepper, virus-induced gene silencing (VIGS) has been used to analyze the functions of structural genes and TFs that are part of the anthocyanin biosynthetic pathway [9, 10, 55, 56]. For example, Zhang et al. (2015) revealed that silencing the R2R3-MYB TF *CaMYB* using VIGS led to the repression of the majority of anthocyanin pathway genes, except for *PAL*, *C4H*, and *4CL* [10]. However, to date, there are no reports yet of the use of combined SLAF-seq and BSA for the study of anthocyanin accumulation in pepper. The present study represents the first application of a combined SLAF-seq/BSA strategy for identification of genomic regions and genes linked to anthocyanin accumulation in pepper.

Sequence data analysis indicated that accurate SLAF libraries were constructed after choosing the appropriate restriction enzyme, sizes of restriction fragments, and evaluating the SLAF libraries against a control library prepared from rice corresponding to our previous study [54]. A total of 771,025 SLAFs evenly distributed along the CM334 reference pepper chromosomes were obtained from the SLAF libraries, and the sequencing depths of SLAFs from both pools and parents were all greater than 20×. For successful SLAF-seq, sequencing depth should exceed 6× and quality scores should be greater than Q30 for [36]. Therefore, our data indicate that we successfully constructed an accurate and high-quality SLAF library for identification of the candidate regions and genes associated with anthocyanin accumulation in pepper.

Molecular markers accurate, high-resolution genetic maps are essential for mapping QTL and for improving the efficiency of marker-assisted selection [57–60]. However, some PCR-based molecular markers such as amplified fragment-length polymorphism (AFLP) [15, 61], random amplified polymorphic DNA (RAPD) [62, 63], and simple sequence repeat (SSR) [32, 64] markers are relatively low-density, non-specific, and provide incomplete coverage. In contrast, SNPs are high frequency markers with denser, genome-wide [65, 66] distributions than SSR or other markers [66]. Their ease of automatic genotyping [67] and high polymorphism make SNPS valuable for molecular genetic analyses [68]. Of the 836,852 SNPs we identified, over 17,073 SNPs were mapped onto the pepper chromosomes, which resulted in denser coverage of the whole pepper genome than in the Cheng et al. (2016) map [69]. Our map will provide higher marker density and increase accuracy for identifying candidate genes. We used a set of 127,004 of high-quality SNPs to perform association analysis and identify candidate anthocyanin-associated regions in pepper.

Overlapping the results of ED-based and SNP-index-based association analyses should improve prediction of candidate regions associated with anthocyanin accumulation, as was done by Geng et al. (2016) for seed weight in *Brassica napus* [31]. SNP-index analysis is more accurate and quantitative for evaluation of frequencies and inheritance of parental alleles in the F_2_ [70]. We narrowed candidate anthocyanin-associated regions down to three within an interval of 14.10 Mb on chromosome 10 that contained 1674 high-quality SNPs. The annotations for these SNPs showed the locations of more than 96% of them in intergenic regions, and would thus be useful for fine-mapping of anthocyanin-related genes.

The pepper CM334 reference genome has been available since 2014 [28], and has allowed comparisons of high-throughput sequencing results from other pepper crosses, which helps to identify polymorphisms throughout the pepper genome. Anthocyanin biosynthetic pathway genes are incompletely dominant and quantitatively inherited in the Solanaceae [15, 23, 71, 72]. Although the enzyme- and TF-encoding genes of the anthocyanin biosynthetic pathway have been extensively studied, most of these genes had not yet been fine mapped in pepper due to the lack of saturated linkage maps. With an accurate and high-quality SLAF library, we identified three candidate regions associated with anthocyanin accumulation on pepper chromosome 10, as did previous reports in pepper [15, 24, 25, 27]. The *A* locus that controls anthocyanin accumulation had previously been mapped to pepper chromosome 10 [24, 25] and found to be allelic to the *fs10*.*1* locus 2.1 cM from the *fs10*.*1* locus [27]. Three candidate regions on chromosome 10 were identified in our study, intervals that ranged from 1.92 to 7.53 Mb in length. Additionally, two adjacent major QTLs (*fap10*.*1*, 106.4 cM and fap10.2, 109.8 cM) [72] associated with control of anthocyanin in eggplant fruit were also mapped to chromosome 10. The *ANT1* and *AN2* loci from tomato have also been mapped to chromosome 10 [73]. A homolog of *AN2* from petunia has also been identified as a candidate gene for the pepper *A* locus [15], and its location corresponds to those of tomato *anthocyanin gainer* and eggplant *fap10*.*1* [71], highlighting the genetic similarities between the solanaceous species pepper, tomato, and eggplant. Therefore, the 14.10-Mb interval on pepper chromosome 10 associated with anthocyanin accumulation is a strong candidate region that could harbor a gene(s) controlling anthocyanin accumulation in pepper.

A series of structural genes that includes *PAL*, *C4H*, *4CL*, *CHS*, *CHI*, *F3H*, *F3’5’H*, *DFR*, *ANS*, *GTs*, *ATs*, and *MTs* [8–10, 15–17], and three regulatory genes including *MYB*, *bHLH* and *WD40* affect anthocyanin biosynthesis [7, 12, 21, 22]. However, there have been limited studies of the genetic regulatory mechanisms underlying the anthocyanin biosynthesis in pepper fruit. Until now, *A* locus is the only one locus to be identified to control the expression of early genes in the pathway, which encoded a CaMYB that cannot control the expression of *PAL*, *C4H*, and *4CL* [10]. The *AN2* has been thought the most likely candidate gene for the pepper *A* locus [15]. Whether there are genes other than *CaMYB* that can control the expression of early structural genes (*PAL*, *C4H*, and *4CL*), and other transcription factors like bHLH and WD40 that can control the anthocyanin accumulation as the candidate genes for bHLH and WD40 have not been found in pepper so far. Besides, Deshpande (1933) also postulated the presence of a second locus other than *A* locus to explain the variation of anthocyanin [74]. In this study, we sought to identify the candidate genes involved in anthocyanin accumulation and variation including *A* locus in pepper fruit. We identified a total of 126 genes in the candidate regions, and 12 of which, annotations indicate, could be related to anthocyanin accumulation. Future studies to isolate and functional testing other genes will likely be aided by the study of these candidate genes. Our results showed that *CA10g04060*, *CA10g03640*, *CA10g0376*, *CA10g03880*, *CA10g12640*, *CA10g12650*, and *CA10g12890* are related to structural genes that might play important roles in pepper fruit anthocyanin biosynthesis. Among these, *CA10g04060* and *CA10g12890* were homologous to *4CL2* from *Nicotiana tabacum* and *CYP76A2* from *Solanum melongena*, respectively, which have been separately related to *4CL* and *F3´5´H*. Shi and Xie (2010) also found that the expression of *4CL2* increased during anthocyanin biosynthesis [50]. *CA10g03640* and *CA10g03760*, which encode 5AT and 3AT respectively, were both predicted to function in acylation of anthocyanin. *CA10g03880*, *CA10g12640*, and *CA10g12650*, homologs of *3GGT*, *UGT73C3*, and *UGT73C5* respectively, have all been annotated as predicted glucosyltransferases. Further, the genes encoding the anthocyanin biosynthetic enzymes are likely also transcriptionally regulated in pepper. We also identified homologs of *MYB39* (*CA10g03650*) and *APL* (*CA10g12810*), which belong to the MYB TF family and could be involved in regulation of anthocyanin metabolism based on the GO annotation. Additionally, we may answer the question rasied by Zhang et al. (2015) that *MYB39* might take part in phenylpropanoid pathway [75]. Zhou et al. (2016) also reported that *APL* took part in phenylpropanoid and flavonoid biosynthesis pathways [52]. *CA10g03650* and *CA10g12810* were different from *A* locus because *A* locus was homologous to tomato *ANT1* and petunia *AN2* [15]. The two genes (*CA10g03650* and *CA10g12810*) might be detected as a second or third gene other than *A* locus to explain the synthesis of anthocyanin, which was consistent with the postulation of Deshpande (1933) [74]. Although previous studies have showed that the method of BSA combined with SLAF-seq in the study was accurate[29,30,32,33,34,54], *A* locus wasn’t identified most possibly because the plant material used in this study was different from that of Borovsky et al. (2004) [15]. At the same time, the function of two genes in anthocyanin metabolism and the relationship between two genes with *CaMYB* need to be further verified. COG annotation indicated that *CA10g12710* is a likely WD40-repeat protein that might regulate the expression of anthocyanin. Finally, *CA10g03810* is related to a gene involved in accumulation of anthocyanin in tissues exposed to ultraviolet light, and a SNP resulting in a non-synonymous mutation in *CA10g12840* was found. All above annotated genes lay a good foundation for the understanding of the genetic regulatory mechanisms of the anthocyanin accumulation in pepper fruit, and allow us to be cloned to further analyze the function of these genes that influence anthocyanin accumulation in this species, but not all genes are identified due to the limitation of BSA and large pepper genome (3.48Gb) as previous study mentioned [54]. Although these genes are homologous to genes that are related to anthocyanin biosynthesis and accumulation, or contain non-synonymous SNPs in pepper, direct evidence as to whether these candidate genes control anthocyanin accumulation in pepper has not been found, but could be revealed by future analyses of the functions of these candidate genes.

## Supporting information

S1 Fig The anthocyanin biosynthesis pathway in pepper. (TIF)

S2 Fig Distribution of SLAFs on the chromosomes of the pepper CM334 reference genome in the restriction enzyme digestion experiment. The *x*-axis and *y*-axis represent chromosome length and chromosome number, respectively. The distance between two adjacent yellow bars indicates 1 Mb on the chromosome, and black lines indicate SLAFs or SNPs.
(TIF)

S3 Fig The distribution of paired-end reads of the rice SLAF control mapped to the rice genome. Fragments between 364 and 414 bp in size were chosen.
(TIF)

S4 Fig Circular graph of sequence variants detected by BSA in pepper. The first to fifth circles in the graph represent, in order, the 12 chromosomes of pepper, gene distribution, SNP density, Euclidean distance values, and ΔSNP-index values related to anthocyanin accumulation.
(TIF)

S5 Fig GO term enrichment analysis of 24 candidate genes related to anthocyanin content according to functional categories in the cellular component, molecular function and biological process domains. (TIF)

S6 Fig Directed acyclic graphs of enriched cellular component domain GO terms in the candidate region associated with anthocyanin accumulation. Each enriched GO term is shown, and the box indicates the 10 most-enriched terms. A detailed description of each GO term and the significance of its enrichment are shown in the box or ellipse. Different colors represent different degrees of significance of enrichment: darker colors indicate greater significance.
(PDF)

S7 Fig Directed acyclic graphs of enriched GO terms from the molecular function domain in the candidate region for anthocyanin accumulation. Each enriched GO term is shown, and the box indicates the 10 most-enriched terms. A detailed description of each GO term and the significance of its enrichment are shown in the box or ellipse. Different colors represent different degrees of significance of enrichment: darker colors indicate greater significance.
(PDF)

S8 Fig Directed acyclic graphs of enriched biological process domain GO terms in the candidate region for anthocyanin accumulation. Each enriched GO term is shown, and the box indicates the 10 most-enriched terms. A detailed description of each GO term and the significance of its enrichment are shown in the box or ellipse. Different colors represent different degrees of significance of enrichment: darker colors indicate greater significance.
(PDF)

S1 Table Anthocyanin concentrations in parental lines and high- and low-anthocyanin pools. (XLSX)

S2 Table The distribution of SLAFs and SNPs on each chromosome of pepper. (DOCX)

S3 Table The properties of all SNPs identified between high- and low-anthocyanin lines and pools. (XLSX)

S4 Table Annotation of SNP markers in the candidate region for high-and low- anthocyanin parents and pools by both Euclidean distance and SNP-index association analysis. (DOCX)

S5 Table Annotation for 126 candidate genes for anthocyanin accumulation. (XLSX)

S6 Table Pathway enrichment analysis via KEGG for candidate genes in pepper. (DOCX)
