# Supplementary figures and images for: Genetic mapping of anthocyanin accumulation-related genes in pepper fruits using a combination of SLAF-seq and BSA
Source: PLoS One. 2018 Sep 27;13(9):e0204690. doi: 10.1371/journal.pone.0204690 (PMC6160195; doi:10.1371/journal.pone.0204690)

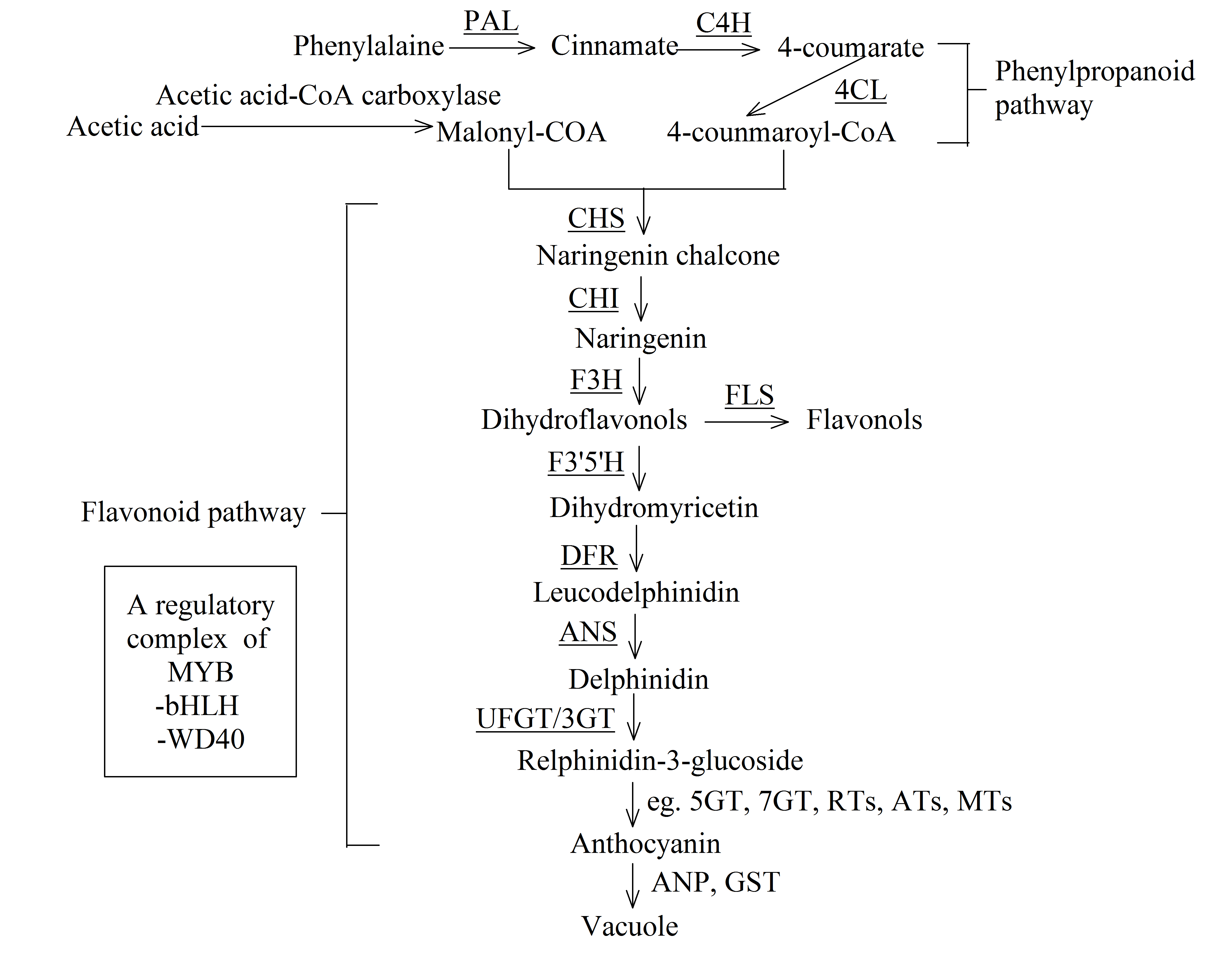

Supplement: S1 Fig — (TIF) [file pone.0204690.s001.tif]

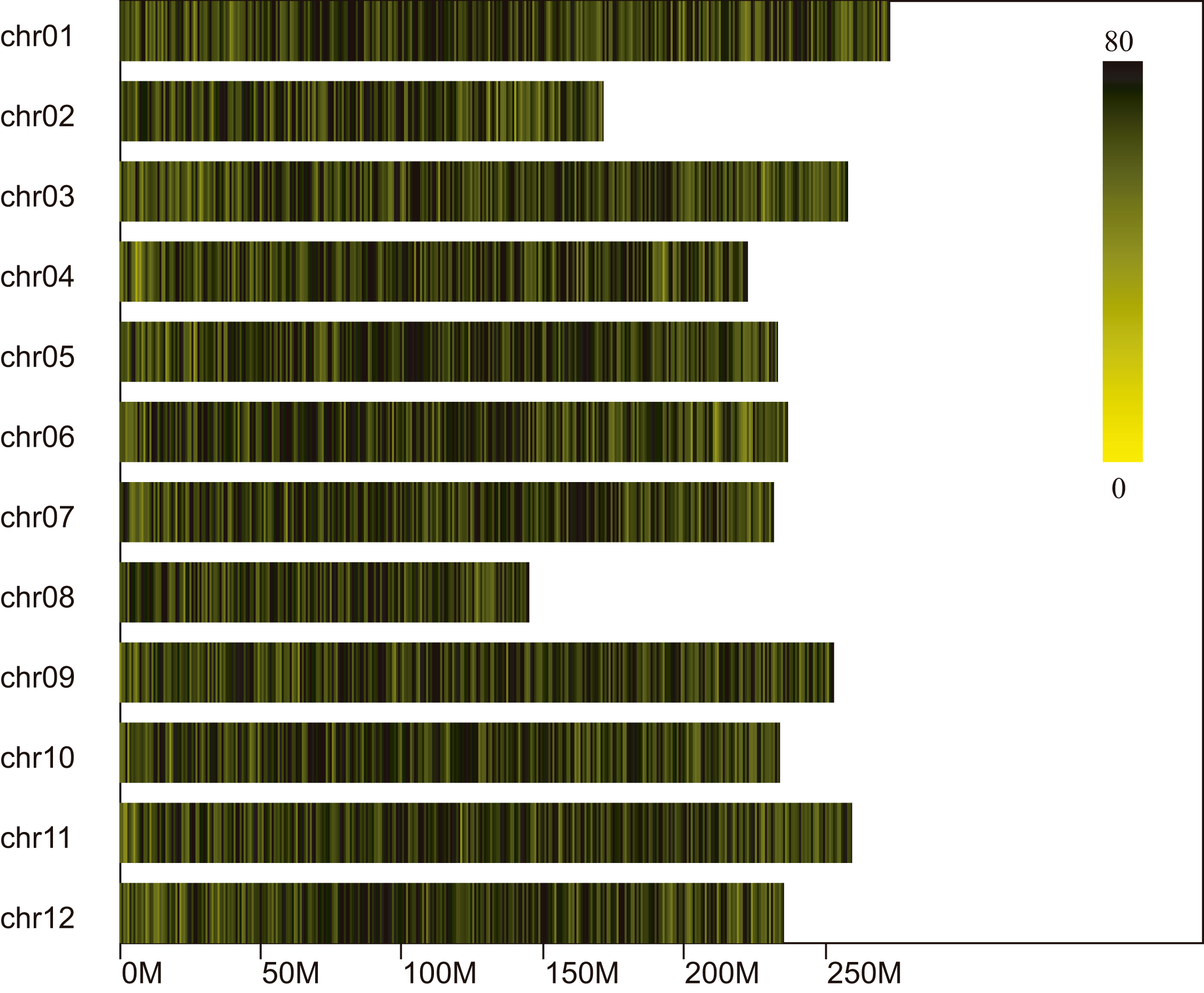

Supplement: S2 Fig — The x-axis and y-axis represent chromosome length and chromosome number, respectively. The distance between two adjacent yellow bars indicates 1 Mb on the chromosome, and black lines indicate SLAFs or SNPs. (TIF) [file pone.0204690.s002.tif]

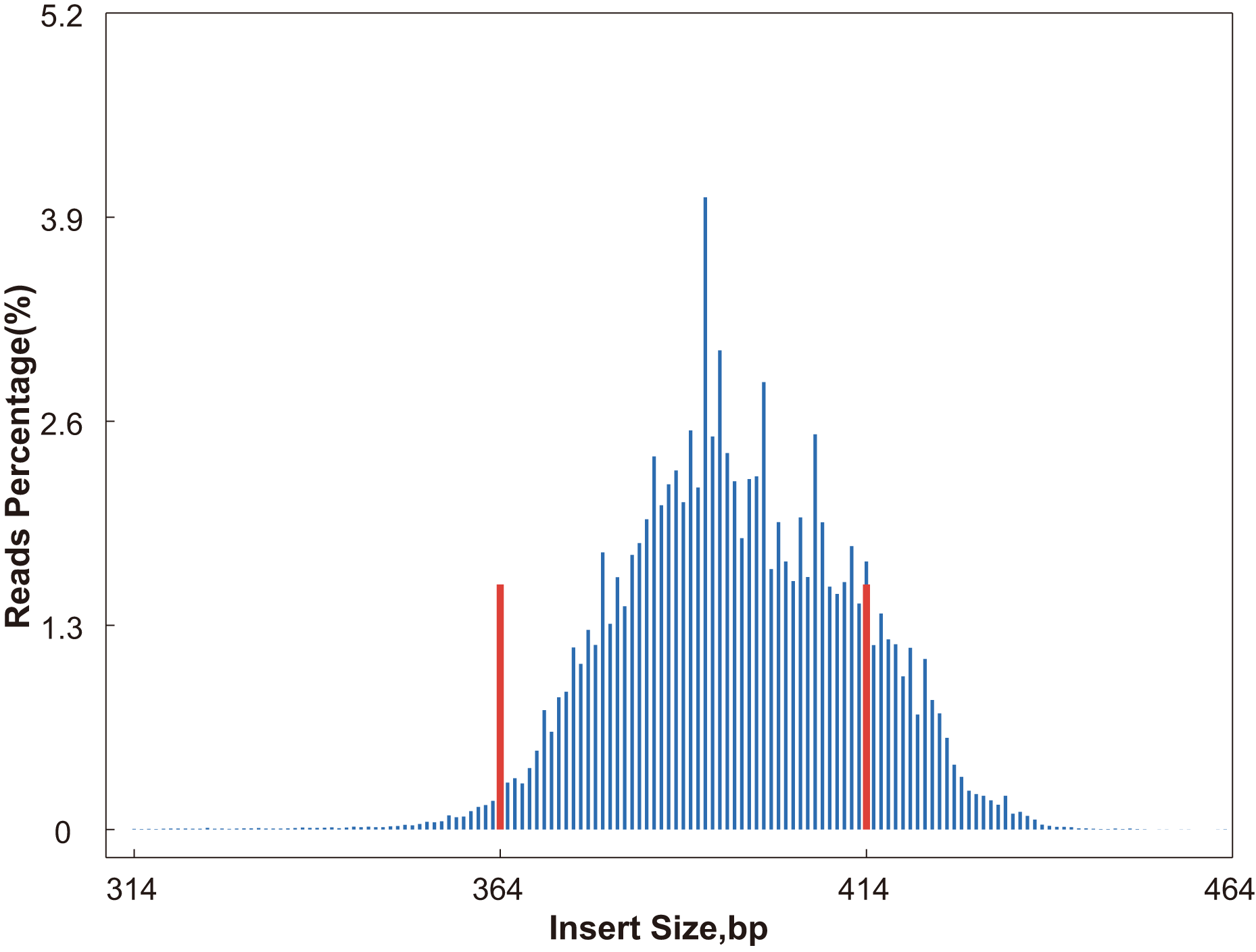

Supplement: S3 Fig — Fragments between 364 and 414 bp in size were chosen. (TIF) [file pone.0204690.s003.tif]

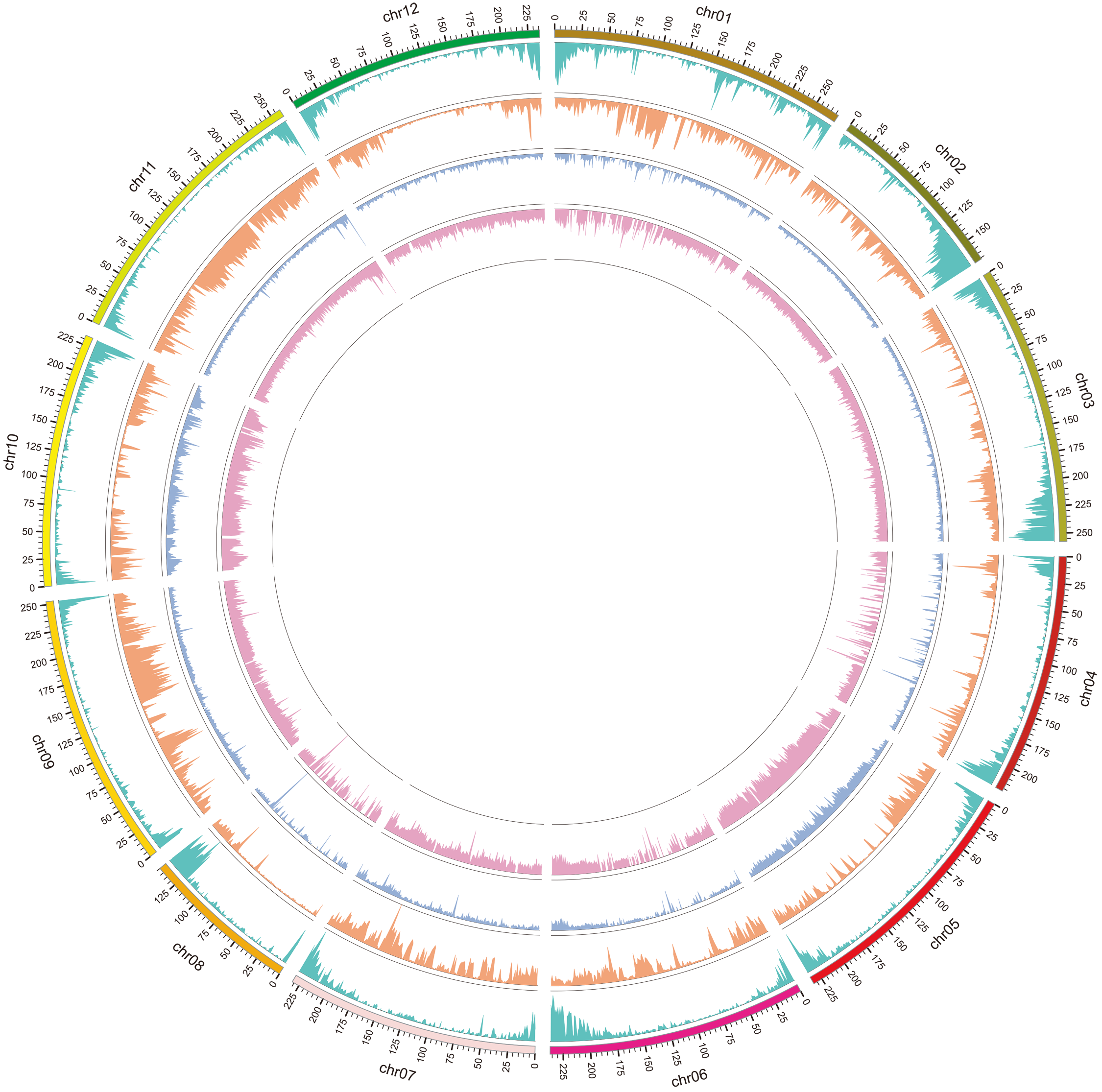

Supplement: S4 Fig — The first to fifth circles in the graph represent, in order, the 12 chromosomes of pepper, gene distribution, SNP density, Euclidean distance values, and ΔSNP-index values related to anthocyanin accumulation. (TIF) [file pone.0204690.s004.tif]

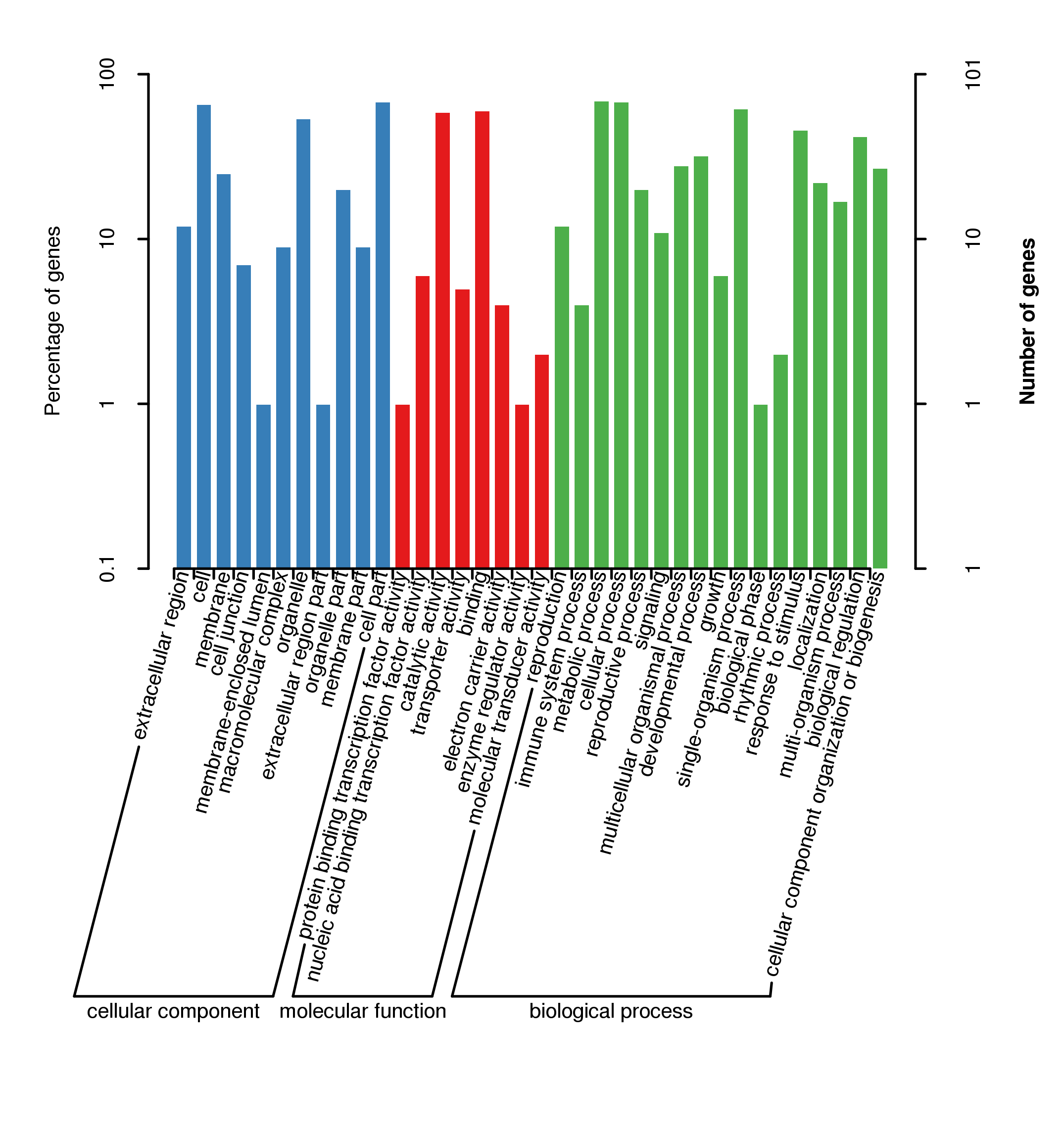

Supplement: S5 Fig — (TIF) [file pone.0204690.s005.tif]

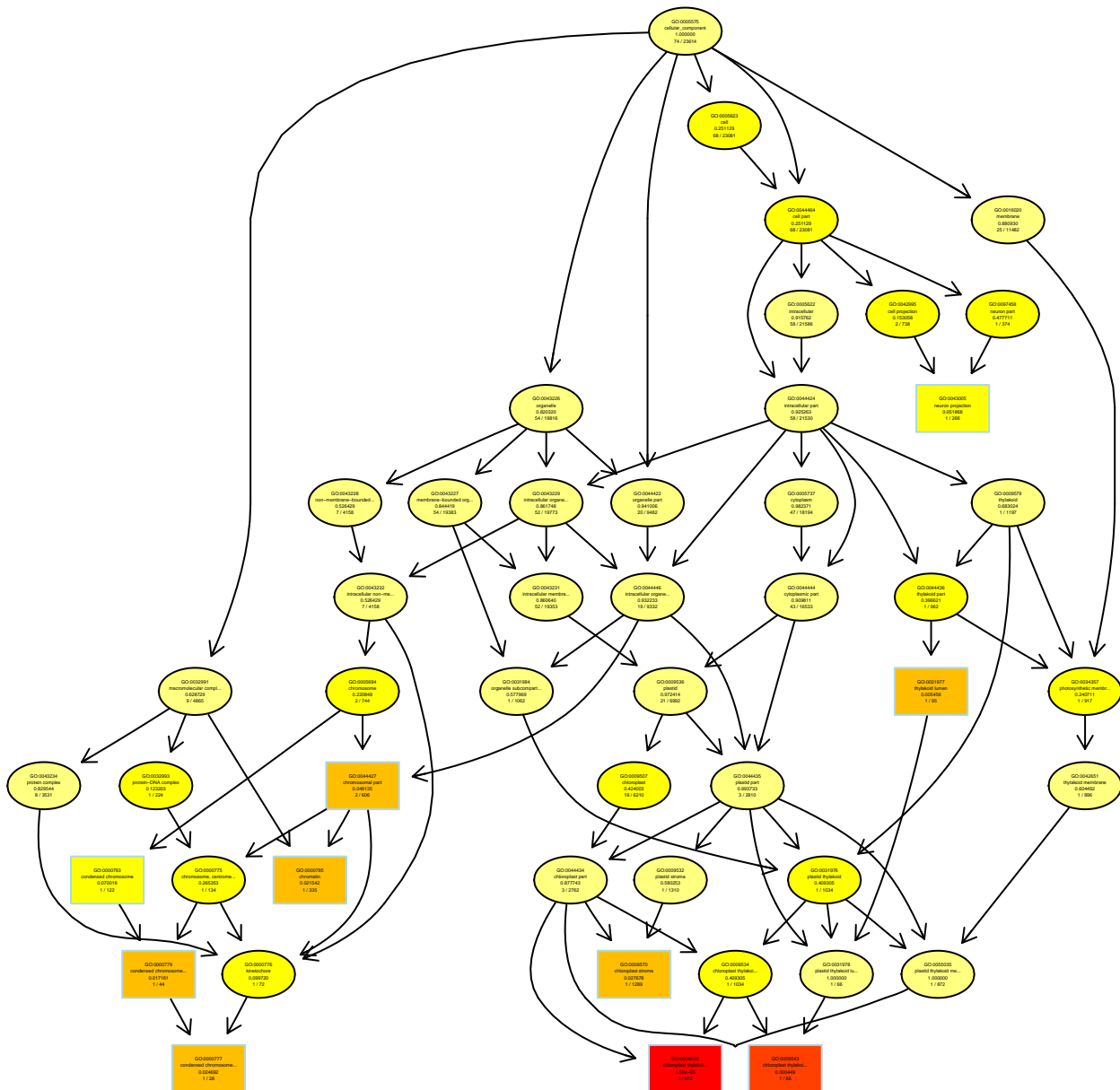

Supplement: S6 Fig — Each enriched GO term is shown, and the box indicates the 10 most-enriched terms. A detailed description of each GO term and the significance of its enrichment are shown in the box or ellipse. Different colors represent different degrees of significance of enrichment: darker colors indicate greater significance. (PDF) [file pone.0204690.s006.pdf]

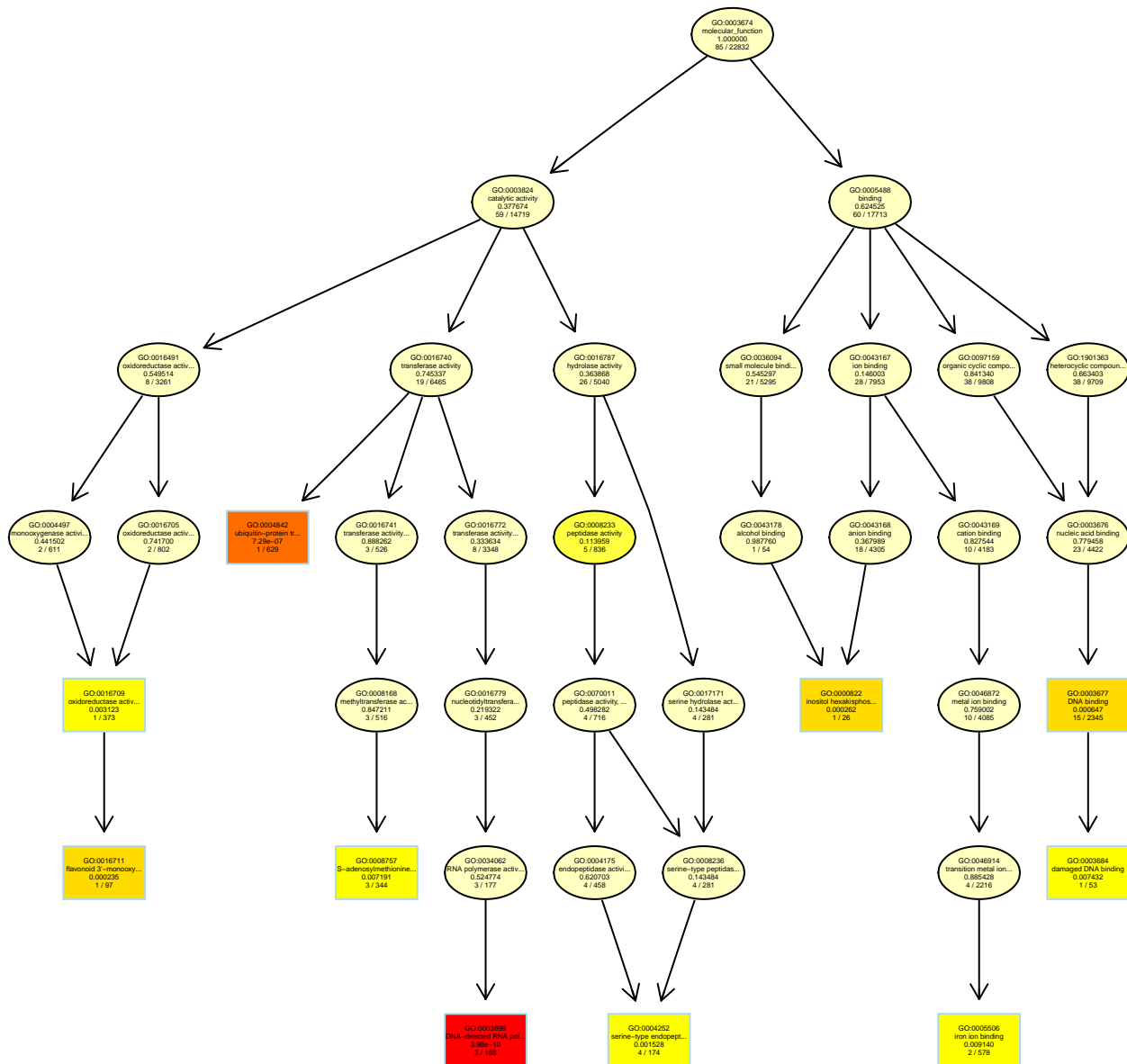

Supplement: S7 Fig — Each enriched GO term is shown, and the box indicates the 10 most-enriched terms. A detailed description of each GO term and the significance of its enrichment are shown in the box or ellipse. Different colors represent different degrees of significance of enrichment: darker colors indicate greater significance. (PDF) [file pone.0204690.s007.pdf]

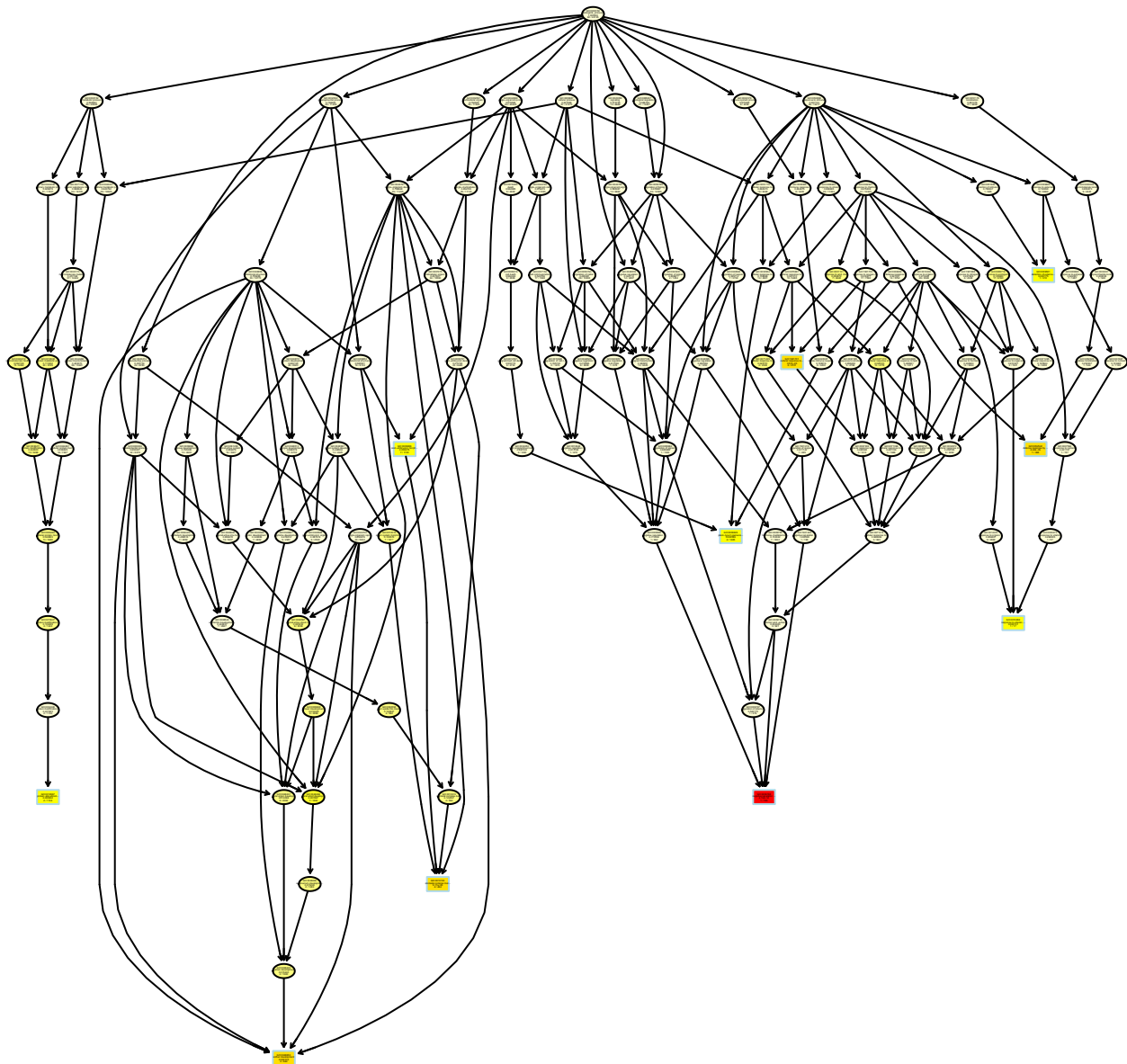

Supplement: S8 Fig — Each enriched GO term is shown, and the box indicates the 10 most-enriched terms. A detailed description of each GO term and the significance of its enrichment are shown in the box or ellipse. Different colors represent different degrees of significance of enrichment: darker colors indicate greater significance. (PDF) [file pone.0204690.s008.pdf]
