# Supplementary material for: Genetic mapping of anthocyanin accumulation-related genes in pepper fruits using a combination of SLAF-seq and BSA
Source: PLoS One. 2018 Sep 27;13(9):e0204690. doi: 10.1371/journal.pone.0204690 (PMC6160195; doi:10.1371/journal.pone.0204690)
Supplement: S2 Table — (DOCX) [file pone.0204690.s010.docx]

**S2 Table. The distribution of SLAFs and SNPs on each chromosome of pepper.**

| **Chromosome ID** | **SLAF number** | **SNP number** |
| --- | --- | --- |
| Chr01 | 73,838 | 93,156 |
| Chr02 | 47,469 | 50,340 |
| Chr03 | 71,160 | 61,320 |
| Chr04 | 61,786 | 42,221 |
| Chr05 | 66,185 | 59,550 |
| Chr06 | 67,529 | 65,789 |
| Chr07 | 65,809 | 81,526 |
| Chr08 | 41,197 | 17,073 |
| Chr09 | 70,461 | 125,376 |
| Ch10 | 67,197 | 65,449 |
| Chr11 | 71,756 | 127,688 |
| Chr12 | 66,638 | 47,364 |
| Total | 771,025 | 836,852 |

SLAF, specific-locus amplified fragment; SNP, single-nucleotide polymorphism.
