# Supplementary material for: Genetic mapping of anthocyanin accumulation-related genes in pepper fruits using a combination of SLAF-seq and BSA
Source: PLoS One. 2018 Sep 27;13(9):e0204690. doi: 10.1371/journal.pone.0204690 (PMC6160195; doi:10.1371/journal.pone.0204690)
Supplement: S4 Table — (DOCX) [file pone.0204690.s012.docx]

**S3 Table. Annotation of SNP markers in the candidate region for high- and low-anthocyanin parents and pools by both Euclidean distance and SNP-index association analysis.**

| **Type** | **Euclidean distance** | | **SNP-index** | |
| --- | --- | --- | --- | --- |
| **Association analysis** | **Z6 vs Z5** | **H-pool vs N-pool** | **Z6 vs Z5** | **H-pool vs N-pool** |
| Intergenic | 7,416 | 2,729 | 2,011 | 894 |
| Intron | 2 | 3 | 2 | 3 |
| Upstream | 48 | 19 | 20 | 10 |
| Downstream | 46 | 23 | 26 | 13 |
| Synonymous_coding | 4 | 3 | 2 | 2 |
| Non_synonymous_coding | 2 | 1 | 1 | 1 |
| Stop_gained | 1 | 1 | 1 | 1 |
| Other | 18 | 2 | 6 | 2 |
| Total | 7,537 | 2,781 | 2,069 | 926 |

H-pool, the pool with the high anthocyanin content; N-pool, the pool with no anthocyanin content.
