# Supplementary material for: Genetic mapping of anthocyanin accumulation-related genes in pepper fruits using a combination of SLAF-seq and BSA
Source: PLoS One. 2018 Sep 27;13(9):e0204690. doi: 10.1371/journal.pone.0204690 (PMC6160195; doi:10.1371/journal.pone.0204690)
Supplement: S6 Table — (DOCX) [file pone.0204690.s014.docx]

**S5 Table. Pathway enrichment analysis via KEGG for candidate genes in pepper.**

| **Pathway** | **KO** | **Enrichment**  **factor** | ***P* value** | **Corrected *P* value** |
| --- | --- | --- | --- | --- |
| RNA polymerase | ko03020 | 6.62 | 0.0095 | 0.038 |
| Homologous recombination | ko03440 | 10.88 | 0.014 | 0.056 |
| Pyrimidine metabolism | ko00240 | 4.37 | 0.029 | 0.12 |
| Purine metabolism | ko00230 | 3.73 | 0.043 | 0.17 |
